# Survey data of COVID-19 awareness, knowledge, preparedness and related behaviors among breast cancer patients in Indonesia

**DOI:** 10.1016/j.dib.2020.106145

**Published:** 2020-08-08

**Authors:** Ricvan Dana Nindrea, Nissa Prima Sari, Wirsma Arif Harahap, Samuel J. Haryono, Hari Kusnanto, Iwan Dwiprahasto, Lutfan Lazuardi, Teguh Aryandono

**Affiliations:** a Doctoral Program, Faculty of Medicine, Public Health and Nursing, Universitas Gadjah Mada, Yogyakarta, Indonesia; b Department of Midwifery, Faculty of Medicine, Universitas Andalas, Padang, Indonesia; c Division of Surgical Oncology, Faculty of Medicine, Universitas Andalas, Padang, Indonesia; d Division of Surgical Oncology, Dharmais Cancer Hospital, Jakarta, Indonesia; e Department of Family and Community Medicine, Faculty of Medicine, Public Health and Nursing, Universitas Gadjah Mada, Yogyakarta, Indonesia; f Department of Pharmacology and Therapy, Faculty of Medicine, Public Health and Nursing, Universitas Gadjah Mada, Yogyakarta, Indonesia; g Department of Health Policy and Management, Faculty of Medicine, Public Health and Nursing, Universitas Gadjah Mada, Yogyakarta, Indonesia; h Department of Surgical Oncology, Faculty of Medicine, Public Health and Nursing, Universitas Gadjah Mada, Yogyakarta, Indonesia

**Keywords:** COVID-19, Breast cancer, Indonesia, Awareness, Knowledge, Preparedness, Behaviors

## Abstract

This dataset presents a survey data describing COVID-19 awareness, knowledge, preparedness and related behaviors among breast cancer patients in Indonesia. The data were collected from breast cancer patients through a survey distributed by an online questionnaire, assesing social-demographic characteristics (6 items), COVID-19 awareness (5 items), knowledge (2 items), preparedness (2 items) and related behaviors (2 items), from 20th June until 14th July 2020. The samples were gathered 500 breast cancer patients in Indonesia who were willing to fill an online questionnaire. SPSS version 23.0 was used to analyzed the data by descriptive and inferential statistics and SmartPLS 3 to created the partial least square path modeling. The data will help in preventing the transmission of COVID-19 among breast cancer patients and can support for health education and promotion interventions.

**Specifications Table**

**Table:** 

**Subject**	Public health
**Specific subject area**	Health education, health promotion
**Type of data**	Primary data Tables Figure
**How data were acquired**	Data was collected using an online survey platform (google forms). The questionnaire is provided as a supplementary file
**Data format**	Raw Analyzed Filtered (descriptive and inferential statistics)
**Parameters for data collection**	The breast cancer patients collected through medical records review at Dr. M. Djamil General Hospital Padang, Sardjito General Hospital Yogyakarta and Dharmais Cancer Hospital Jakarta**.** The survey data was conducted from 500 breast cancer patients in Indonesia to assesing COVID-19 awareness, knowledge, preparedness and related behaviors with internet access.
**Description of data collection**	The survey data was conducted through an online questionnaire, which was delivered to breast cancer patients in Indonesia with convenience sampling technique.
**Data source location**	Region: Southeast Asia Country: Indonesia
**Data accessibility**	The data are available in Mendeley Data https://data.mendeley.com/datasets/th4k22mf4f/draft?a=f9071e1a-d39d-4983-9350-ada62261d845

**Value of the Data**

• These data are useful because this is the first survey that involved 500 of respondents that explore COVID-19 awareness, knowledge, preparedness and related behaviors among breast cancer patients in Indonesia.
• All researchers in epidemiology, cancer registry, and health psychology can benefit from these data because by using this data to give the government recommendations to help in preventing the spread of COVID-19 among breast cancer patients and can support for health education and promotion interventions in their country.
• The data will be valuable to researchers who want to compare with similar studies on COVID-19 awareness, knowledge, preparedness and related behaviors among breast cancer patients from other countries or developing to systematic review and also meta-analysis in the future
• These data could potentially make an impact on society, involving other variables that influence of breast cancer patients behaviors to prevent the transmission of COVID-19.

## 1 Data description

The dataset provides an insightful information based on survey data on COVID-19 awareness, knowledge, preparedness and related behaviors among breast cancer patients in Indonesia. The breast cancer patients collected through medical records review at Dr. M. Djamil General Hospital Padang, Sardjito General Hospital Yogyakarta and Dharmais Cancer Hospital Jakarta**.** The survey data was conducted from 500 breast cancer patients in Indonesia to assesing COVID-19 awareness, knowledge, preparedness and related behaviors with internet access. The data include five major group of variable: (a) social-demographic characteristics, including age, educational background, working status, marital status and nutritional status; (b) Five items for COVID-19 awareness including information about COVID-19, seriousness of COVID-19, COVID-19 as a public health threats, probability get sick from COVID-19 and someone arround of participants get COVID-19; (c) two items assesed COVID-19 related to knowledge including correctly identified 3 symptoms of the COVID-19 and correctly identified 3 prevention methods of the COVID-19; (d) two items measured COVID-19 related to preparedness government confident to prevent of COVID-19 and preparedness related to COVID-19 outbreak; (e) two items assesed their COVID-19 related behaviors including COVID-19 changed daily routine and plans. The questionnaire is provided as a supplementary file. Social-demographic characteristics are presented in Table 1.

**Table 1:** Social-demographic characteristics (*n* = 500).

Characteristics	Category	f (%)
*Age (years)*	< 50	321 (64.2)
	≥ 50	179 (35.8)
*Educational background*	No school	5 (1.0)
	Elementary school	48 (9.6)
	Junior high school	41 (8.2)
	Senior high school	221 (44.2)
	Bachelor degree	170 (34.0)
	Master degree	15 (3.0)
*Working status*	Housewife	303 (60.6)
	Civil servant	146 (29.2)
	Private servant	32 (6.4)
	Enterpreneur	2 (0.4)
	Farmer	7 (1.4)
	Retired	8 (1.6)
	Laborer	2 (0.4)
*Marital status*	Single/ widow	19 (3.8)
	Marriage	481 (96.2)
*Nutritional status*	Normal	210 (42.0)
	Overweight	87 (17.4)
	Obese	203 (40.6)

**Fig. 1:**
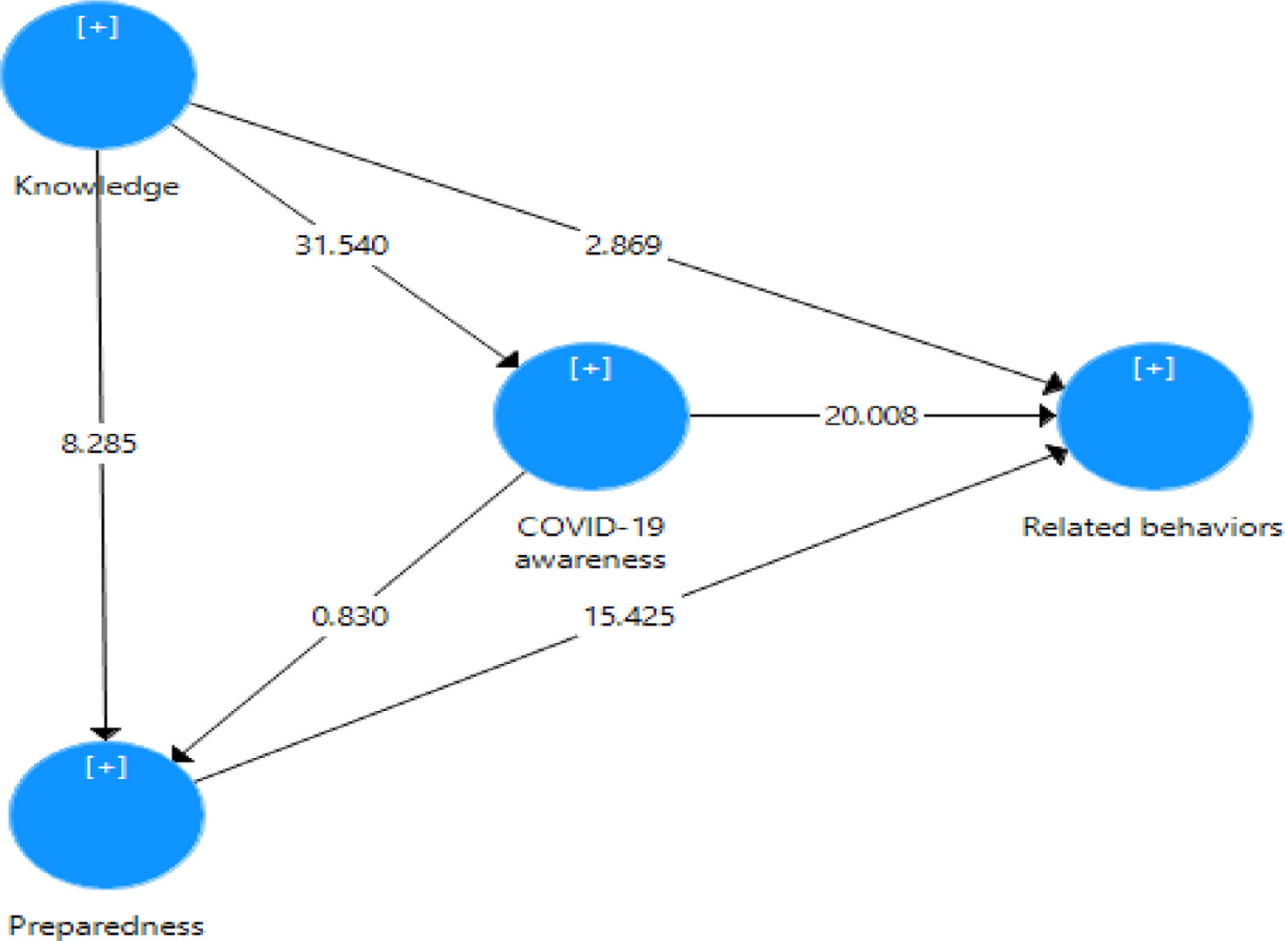
Partial least square path modeling COVID-19 awareness, knowledge, preparedness and related behaviors among breast cancer patients in Indonesia.

The detailed measurement of responses on COVID-19 awareness, knowledge, preparedness and related behaviors among breast cancer patients in Indonesia are described in Tables 2–5. Correlation between COVID-19 awareness, knowledge, preparedness and related behaviors among breast cancer patients In Indonesia are described in Table 6. Partial least square path modeling COVID-19 awareness, knowledge, preparedness and related behaviors among breast cancer patients In Indonesia in Fig. 1.

**Table 2:** Responses to COVID-19 awareness among breast cancer patients in Indonesia.

COVID-19 awareness	Answer	f (%)
How worried are you about getting the COVID-19?	Not worried at all	2 (0.4)
	A little worried	6 (1.2)
	Somewhat worried	251 (50.2)
	Very worried	241 (48.2)
How worried are you about getting the flu	Not worried at all	2 (0.4)
	A little worried	6 (1.2)
	Somewhat worried	387 (77.4)
	Very worried	105 (21.0)
Did you get a flu shot this past year?	No	29 (5.8)
	Yes	471 (94.2)
Do you think that you will get sick from the COVID-19	Not at all	5 (1.0)
	Its possible	9 (1.8)
	I probably will	331 (66.2)
	I definitely will	155 (31.0)
How likely do you think it is that you or someone you know may get sick from COVID-19 this year	Not at all likely	3 (0.6)
	Not that likely	10 (2.0)
	Somewhat likely	275 (55.0)
	Very likely	212 (42.4)

**Table 3:** Responses to knowledge about COVID-19 among breast cancer patients in Indonesia.

Knowledge about COVID-19	Answer	f (%)
Correctly identified 3 symptoms of the COVID-19	No	288 (57.6)
	Yes	212 (42.4)
Correctly identified 3 prevention methods of the COVID-19	No	264 (52.8)
	Yes	236 (47.2)

**Table 4:** Responses to preparedness about COVID-19 among breast cancer patients in Indonesia.

Knowledge to preparedness about COVID-19	Answer	f (%)
How confident are you that the government can prevent a nationwide outbreak at the COVID-19	Not confident at all	186 (37.2)
	Not very confident	314 (62.8)
	Somewhat confident	0
	Very confident	0
How prepared do you think you are if there were to be a widespread COVID-19 outbreak	Not prepared	186 (37.2)
	A little prepared	163 (32.6)
	Somewhat prepared	151 (30.2)
	Very prepared	0

**Table 5:** Responses to behaviors related to COVID-19 among breast cancer patients in Indonesia.

Behaviors related to COVID-19	Answer	f (%)
How much has the COVID-19 change your daily routine?	Not at all	0
	A little	43 (8.6)
	Some	309 (61.8)
	A lot	148 (29.6)
Are you changing any plans that you have made because at the COVID-19	No	148 (29.6)
	Yes	352 (70.4)

**Table 6:** Correlation between COVID-19 awareness, knowledge, preparedness and related behaviors among breast cancer patients In Indonesia.

Variable	*R* value	*p*-value
COVID-19 awareness – knowledge	0.731	<0.001
COVID-19 awareness – preparedness	0.386	<0.001
COVID-19 awareness – related behaviors	0.820	<0.001
Knowledge – preparedness	0.489	<0.001
Knowledge – related behaviors	0.727	<0.001
Preparedness – related behaviors	0.658	<0.001

## 2 Experimental design, materials and methods

This research was conducted using a cross sectional survey design to determine COVID-19 awareness, knowledge and preparedness with related behaviors among breast cancer patients in Indonesia. The dataset in this survey were 500 breast cancer patients collected through medical records review at Dr. M. Djamil General Hospital Padang, Sardjito General Hospital Yogyakarta and Dharmais Cancer Hospital Jakarta, by the written online informed consent. The data responses collected between 20th June until 14th July 2020. The main researchers selected to use WhatsApp Messenger for enrolling potential participants. A questionnaire was designed and executed and made using google forms and link generated was shared on Whatsapp messenger after main researchers got the contact number of participants from medical records review and permitted by doctors or team members who treated patients at Dr. M. Djamil General Hospital Padang, Sardjito General Hospital Yogyakarta and Dharmais Cancer Hospital Jakarta. The sampling technique in this survey is convenience sampling [1]. The inclusion criteria were female patients with pathology examination showed positive breast cancer based on medical records review and never infected COVID-19 [2].

The survey items of COVID-19 awareness were adapted used previous studies [3,4], knowledge preparedness, behaviors related to COVID-19 questionnaire items were adapted from previous study by Wolf et al. [4]. The questionnaire translating to Indonesian.

The respondent's social-demographics analyzed using frequency and percentage. The COVID-19 awareness, knowledge, preparedness and related behaviors among breast cancer patients analyzed using Pearson correlation test. *P* value < 0.05 was stated as statistically significant.

## Ethics statement

This study passed the ethical review by the ethics commiittee of the Faculty of Medicine, Public Health and Nursing, Universitas Gadjah Mada, Yogyakarta, Indonesia. The survey data was conducted according to the Declaration of Helsinki.

## Declaration of Competing Interest

The authors declare that they have no known competing financial interests or personal relationships which have, or could be perceived to have, influenced the work reported in this article.

## Appendix Supplementary materials

Image, application 1
