# Supplementary material for: Survey data of COVID-19 awareness, knowledge, preparedness and related behaviors among breast cancer patients in Indonesia
Source: Data Brief. 2020 Aug 8;32:106145. doi: 10.1016/j.dib.2020.106145 (PMC7413840; doi:10.1016/j.dib.2020.106145)
Supplement: Supplementary file 1 [file mmc1.doc]

**Supplementary materials**

**Table 1. The questionnaire of COVID-19 awareness knowledge, preparedness and related behaviors among breast cancer patients**

| **Questions*** | **Answer** |
| --- | --- |
| **A. COVID-19 awareness** |  |
| A1. How worried are you about getting the COVID-19? | Not worried at all |
|  | A little worried |
|  | Somewhat worried |
|  | Very worried |
| A2. How worried are you about getting the flu | Not worried at all |
|  | A little worried |
|  | Somewhat worried |
|  | Very worried |
| A3. Did you get a flu shot this past year? | No |
|  | Yes |
| A4. Do you think that you will get sick from the COVID-19 | Not at all |
|  | Its possible |
|  | I probably will |
|  | I definitely will |
| A5. How likely do you think it is that you or someone you know may get sick from COVID-19 this year | Not at all likely |
|  | Not that likely |
|  | Somewhat likely |
|  | Very likely |
| **B. Knowledge about COVID-19** |  |
| B1. Correctly identified 3 symptoms of the COVID-19 | No |
|  | Yes |
| B2. Correctly identified 3 prevention methods of the COVID-19 | No |
|  | Yes |
| **C. Preparedness about COVID-19** |  |
| C1. How confident are you that the government can prevent a nationwide outbreak at the COVID-19 | Not confident at all |
|  | Not very confident |
|  | Somewhat confident |
|  | Very confident |
| C2. How prepared do you think you are if there were to be a widespread COVID-19 outbreak | Not prepared |
|  | A little prepared |
|  | Somewhat prepared |
|  | Very prepared |
| **D. Behaviors related to COVID-19** |  |
| D1. How much has the COVID-19 change your daily routine? | Not at all |
|  | A little |
|  | Some |
|  | A lot |
| D2. Are you changing any plans that you have made because at the COVID-19 | No |
|  | Yes |

*M.S. Wolf, M. Serper, L. Opsasnick, R.M. O’Conor, L.M. Curtis, J.Y. Benavente, et al, Awareness, attitudes, and actions related to COVID-19 among adults with chronic conditions at the onset of the U.S. outbreak: a cross-sectional survey, Ann. Intern. Med. 2020 (2020), M20-1239
